# O-Specific Antigen-Dependent Surface Hydrophobicity Mediates Aggregate Assembly Type in Pseudomonas aeruginosa

**DOI:** 10.1128/mBio.00860-21

**Published:** 2021-08-10

**Authors:** Sheyda Azimi, Jacob Thomas, Sara E. Cleland, Jennifer E. Curtis, Joanna B. Goldberg, Stephen P. Diggle

**Affiliations:** a Center for Microbial Dynamics and Infection, School of Biological Sciences, Georgia Institute of Technology, Atlanta, Georgia, USA; b School of Physics, Georgia Institute of Technology, Atlanta, Georgia, USA; c Division of Pulmonology, Allergy/Immunology, Cystic Fibrosis and Sleep, Department of Pediatrics, Emory University School of Medicine, Atlanta, Georgia, USA; University of Georgia

**Keywords:** lipopolysaccharide, O-antigen, *Pseudomonas aeruginosa*, cystic fibrosis, hydrophobicity, depletion aggregation

## Abstract

Bacteria live in spatially organized aggregates during chronic infections, where they adapt to the host environment, evade immune responses, and resist therapeutic interventions. Although it is known that environmental factors such as polymers influence bacterial aggregation, it is not clear how bacterial adaptation during chronic infection impacts the formation and spatial organization of aggregates in the presence of polymers. Here, we show that in an *in vitro* model of cystic fibrosis (CF) containing the polymers extracellular DNA (eDNA) and mucin, O-specific antigen is a major factor determining the formation of two distinct aggregate assembly types of Pseudomonas aeruginosa due to alterations in cell surface hydrophobicity. Our findings suggest that during chronic infection, the interplay between cell surface properties and polymers in the environment may influence the formation and structure of bacterial aggregates, which would shed new light on the fitness costs and benefits of O-antigen production in environments such as CF lungs.

## INTRODUCTION

During chronic infection, biofilm-forming cells are often more tolerant to antibiotics and the host immune response than planktonic cells (1–3). Biofilms also allow individual cells the physical proximity to engage in and benefit from social behaviors such as quorum sensing (QS) and the production of secreted common goods (4–9). Biofilms formed during infection often take the form of cellular aggregates (8, 10–12). In the fluids of wounds and airways of cystic fibrosis (CF) patients, the opportunistic pathogen Pseudomonas aeruginosa frequently grows as freely suspended aggregates of ∼10 to 10,000 cells (6, 8, 10, 11). The mechanisms that govern the shape and size of bacterial aggregates during infection are not well defined. In polymer-rich environments, aggregates have been shown to form by either (i) an increase in entropic force (depletion aggregation) (13, 14) or (ii) electrostatic interactions between bacterial cell surfaces and polymers in the environment (bridging aggregation) (15–18).

P. aeruginosa populations become phenotypically and genetically diverse over time in the complex microenvironments found in CF airways (19–22), and the importance of this heterogeneity for the formation and organization of aggregates has not been assessed. To resolve this, we evaluated aggregate formation in seven genetically diverse isolates sourced from heterogeneous populations of the P. aeruginosa strain PAO1 previously evolved in biofilms for 50 days (23). We assessed how each isolate formed aggregates in a polymer-enriched, spatially structured CF growth medium (SCFM2; containing mucin and extracellular DNA [eDNA] polymers), which has previously been used as an *in vitro* CF model to study the biogeography and physiology of P. aeruginosa (8, 24–26).

We found that the PAO1 ancestor and five isolates formed a stacked pattern (stacked aggregates), where cells closely packed lengthwise, similar to those identified in previous polymer-driven depletion-aggregation studies (27). Two isolates formed distinct disorganized aggregates of various sizes (clumped aggregates), similar to aggregates previously observed in CF sputum samples (10, 28). Whole-genome sequencing showed that the two clumping isolates had alterations in the *ssg* gene (PA5001), which has previously been shown to be involved in lipopolysaccharide (LPS) core and O-antigen biosynthesis (29–32). In P. aeruginosa, LPS contains three major components: the lipid A layer of the outer membrane, a core oligosaccharide, and O-antigen components. O-antigens are further subdivided into a d-rhamnose homopolymer found in most strains called the common polysaccharide antigen (CPA) (formerly A-band) and a variable heteropolymer of 3 to 5 sugars called the O-specific antigen (OSA) (formerly B-band) that confers serotype specificity (29, 32).

We hypothesized that changes in O-antigen glycoforms capping LPS would lead to different aggregate assembly types by altering the physicochemical properties of the bacterial cell surface. This could result in new interactions (e.g., between the bacteria or the bacteria and polymers) that compete with the entropic force that otherwise leads to stacked aggregation in this polymer-rich environment. To elucidate the contribution of LPS-capping glycoforms to aggregate assembly, we assessed the aggregate formation of clean deletion mutants of OSA and CPA. We found that the loss of OSA and changes in the capped LPS core glycoforms led to increased hydrophobicity of the cell surface that overcame the entropic force imposed by host polymers, resulting in disorganized, irreversible clumping. Most importantly, we demonstrate that aggregate shape and structure are dependent on the interplay between the physical properties of the environment and the biological mediation of bacterial cell surface properties governed by the LPS core and OSA.

## RESULTS

### Distinct aggregate assembly types in genetically diverse P. aeruginosa isolates.

**FIG 1:**
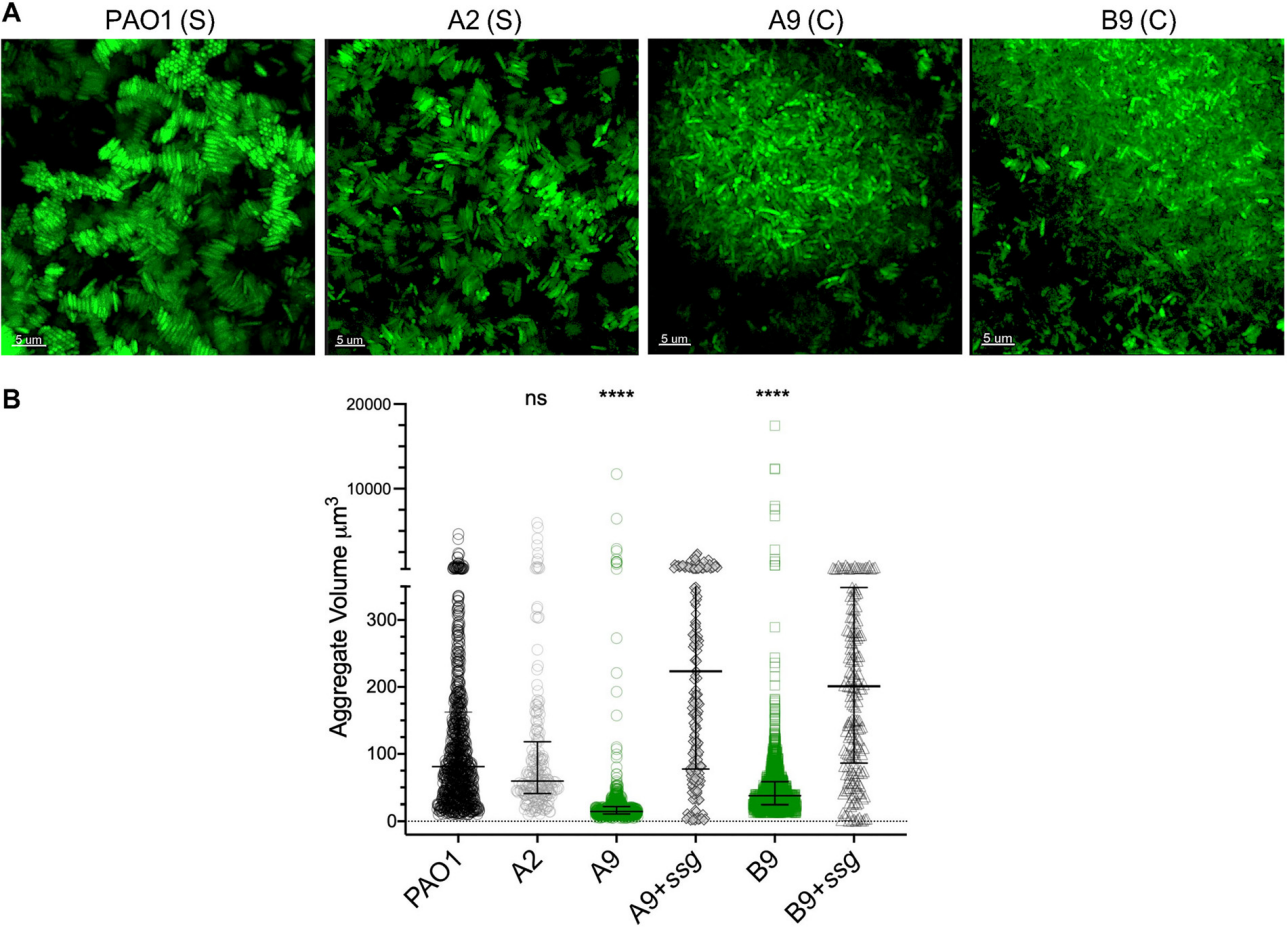
The two types of aggregate assemblies formed by P. aeruginosa isolates in SCFM2 are due to *ssg* gene mutation. (A) In PAO1 and evolved isolates, aggregates assembled into either organized stacked structures (labeled S) or disorganized clumps (labeled C). (B) Stacked aggregates of PAO1 and A2 were significantly larger than aggregates formed by A9 and B9, and complementation with an intact *ssg* gene significantly increased the aggregate volume (*P < *0.0001 by Kruskal-Wallis and Dunn’s multiple-comparison tests; error bars are median aggregate volumes with interquartile ranges, and each data point is representative of an aggregate). ns, not significant.

Genetically and morphologically heterogeneous isolates of P. aeruginosa are commonly collected from expectorated CF sputum samples (20, 21, 33, 34). Since it is known that several lineages of P. aeruginosa can stably coexist in CF airways, we tested whether population heterogeneity impacted aggregate formation. We chose seven distinct morphotypes isolated from a previous study where we evolved biofilms of PAO1 in synthetic polymer-free sputum medium (SCFM) for 50 days (23, 35, 36) (see Fig. S1A in the supplemental material). We assessed aggregate formation in a spatially structured iteration of SCFM termed SCFM2, which contains mucin and eDNA polymers (24). We identified two distinct types of aggregate assembly where PAO1 and five of the evolved isolates (A2, B8, B13, C25, and D4) were assembled into stacked aggregates, where cells were closely aligned side by side by entropic force, similar to previous reports (27) (Fig. 1A; Fig. S1B and C). In contrast, two of the evolved isolates (A9 and B9) formed clumped aggregates that appeared as disorganized small and large groups of cells, similar to bridging aggregation (15) (Fig. 1A). We also investigated the growth of A9 and B9 in SCFM (no addition of eDNA or mucin) and observed that both strains formed clumps even in a polymer-free environment, while the other isolates did not form any aggregates (Fig. S2A).

10.1128/mBio.00860-21.1 FIG S1 Aggregate assembly of PAO1 evolved isolates in SCFM2. (A) Evolved isolates of PAO1 displayed differential colony morphologies on Congo red agar plates. (B) Evolved isolates B8, B13, C25, and D4 displayed stacked aggregate assemblies (labeled S). (C) There were no significant differences in the volumes of stacked aggregates in these isolates compared to PAO1 (*P* < 0.0001 by Kruskal-Wallis and Dunn’s multiple-comparison tests; error bars are median aggregate volumes with interquartile ranges). Download FIG S1, TIF file, 2.6 MB. Copyright © 2021 Azimi et al. 2021 Azimi et al. https://creativecommons.org/licenses/by/4.0/ This content is distributed under the terms of the Creative Commons Attribution 4.0 International license.

10.1128/mBio.00860-21.2 FIG S2 Mutation in PA5001 (*ssg*) in PAO1 switches the stacked aggregate assembly to a clumped assembly. (A) PAO1 and A2 cells formed a dispersed layer of cells on coverslips when grown in SCFM (without any polymer), while A9 and B9 formed small clumps in SCFM. (B) Complementing A9 and B9 isolates with *ssg* restored the aggregate assembly to the stacked type (*P < *0.0001 by Kruskal-Wallis and Dunn’s multiple-comparison tests; error bars are median aggregate volumes with interquartile ranges). Download FIG S2, TIF file, 1.1 MB. Copyright © 2021 Azimi et al. 2021 Azimi et al. https://creativecommons.org/licenses/by/4.0/ This content is distributed under the terms of the Creative Commons Attribution 4.0 International license.

To identify the genetic determinants of clumping assembly in the evolved isolates, we performed whole-genome sequencing on each isolate using the Illumina MiSeq platform. We used breseq (0.34.0) for variant calling between the evolved isolates and the PAO1 ancestor (37). While we found differential levels of genetic variation in each isolate compared to PAO1, we observed that A9 and B9 each contained a 1-bp deletion in the *ssg* gene (PA5001) (Data Set S1). To confirm that mutation of *ssg* results in clumped aggregate assembly, we complemented A9 and B9 with an intact *ssg* gene in *trans*. We found that in both isolates, *ssg* complementation restored the stacked aggregate assembly seen in PAO1 (Fig. 1B; Fig. S2B), suggesting that Ssg plays a role in the aggregate assembly type.

10.1128/mBio.00860-21.7 DATA SET S1 List of SNPs in 7 evolved isolates of PAO1. Download Data Set S1, XLSX file, 0.03 MB. Copyright © 2021 Azimi et al. 2021 Azimi et al. https://creativecommons.org/licenses/by/4.0/ This content is distributed under the terms of the Creative Commons Attribution 4.0 International license.

A distinct feature of the stacked versus clumped aggregates was the average volume. We found that stacked aggregate volumes were 2 to 4 times larger (median aggregate sizes of ∼63 μm^3^ and 89 μm^3^ for PAO1 and A2, respectively, and ∼200 μm^3^ for *ssg*-complemented A9 and B9) than those of clumped aggregates (median aggregate sizes of ∼23 and 30 μm^3^ for A9 and B9, respectively) (Fig. 1B). To quantify these observed differences in the distribution of aggregate biomass in cells with stacked and clumped aggregate assembly types, we compared the distributions of biovolume (ratio of the aggregate volume to the surface area) for each type of aggregate assembly in SCFM2. We found that regardless of the size, the median biovolume in stacked aggregates was significantly larger than that in clumped aggregates (Table S1).

10.1128/mBio.00860-21.5 TABLE S1 Stacked aggregates have higher biovolumes than clumped aggregates. To determine the differences in biomass of each aggregate type, we calculated the total biovolume (cubic micrometers/square micrometers) of all aggregates in each acquired image using Imaris. There was a significant difference between the distributions of biovolumes of stacked and clumped (green) aggregates (*P < *0.0001 by Kruskal-Wallis and Dunn’s multiple-comparison tests; error bars are median aggregate biovolumes with interquartile ranges). Download Table S1, DOCX file, 0.01 MB. Copyright © 2021 Azimi et al. 2021 Azimi et al. https://creativecommons.org/licenses/by/4.0/ This content is distributed under the terms of the Creative Commons Attribution 4.0 International license.

### OSA and not other biofilm traits determines aggregate assembly type.

**FIG 2:**
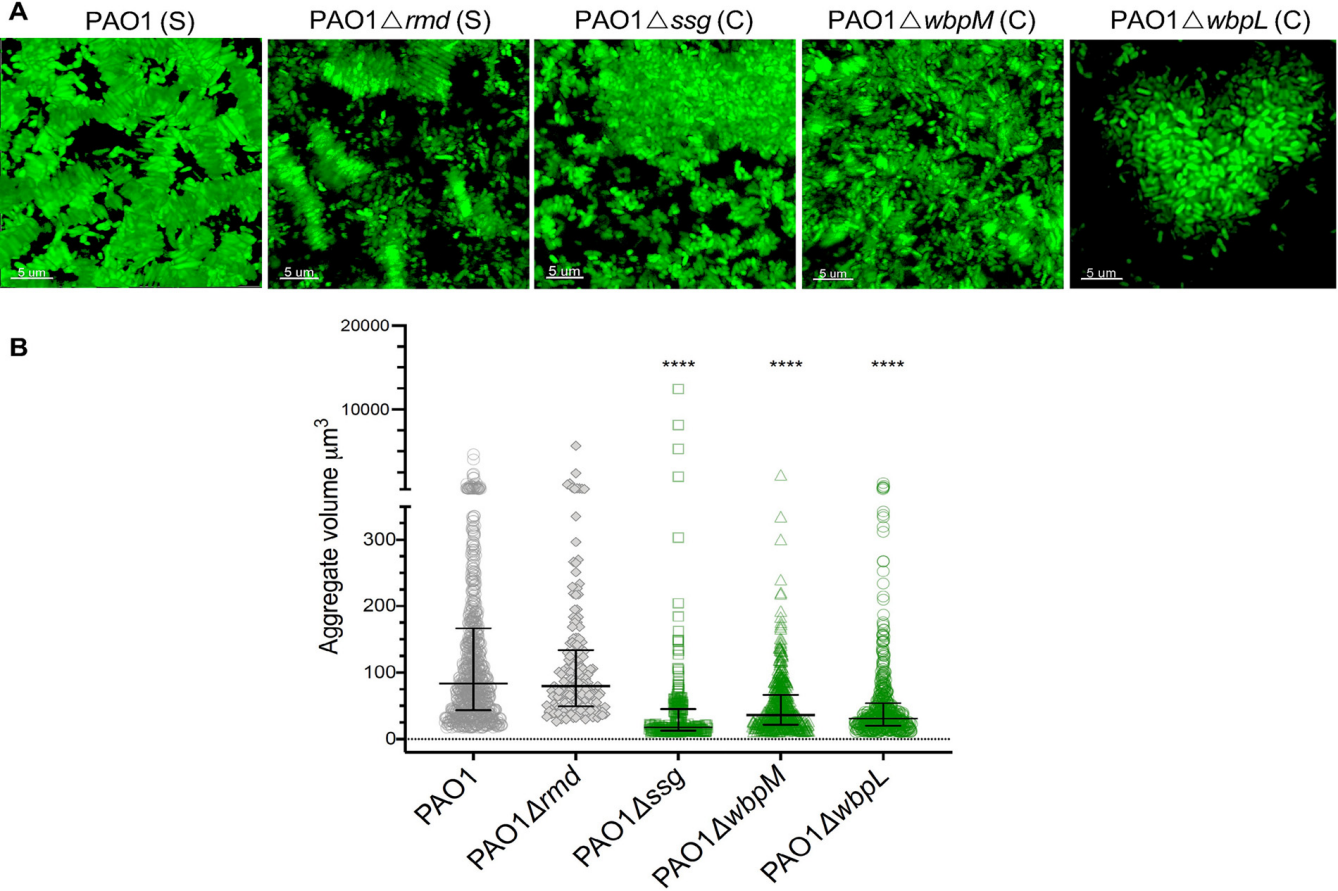
Loss of OSA leads to clumped aggregate assembly. (A) The loss of CPA (Δ*rmd*) did not alter the type of aggregate assembly, and the loss of OSA (Δ*wbpM*) led to dispersed small aggregates. The loss of both CPA and OSA (Δ*wbpL*) changed the aggregate assembly type similarly to the *ssg* mutant. (B) There was a significant reduction in the aggregate volume in *ssg*, *wbpL*, and *wbpM* mutants, but only the loss of *ssg* and *wbpL* displayed large, clumped aggregates (*P < *0.0001 by Kruskal-Wallis and Dunn’s multiple-comparison tests; error bars are median aggregate volumes with interquartile ranges, and each point is representative of an aggregate).

The proposed function of Ssg is a glycosyltransferase, involved in LPS and exopolysaccharide (EPS) biosynthesis (30, 38). P. aeruginosa strains with mutations in *ssg* have previously been shown to display decreased motility, enhanced phage resistance, and a lack of O-antigen (30, 31, 39). We next determined whether the different aggregate assemblies (due to the loss of *ssg* in our evolved PAO1 isolates) were because of differences in O-antigen production. We constructed a clean *ssg* gene deletion in PAO1 (PAO1 Δ*ssg*) and a range of isogenic LPS synthesis or O-antigen assembly mutants. These were (i) mannose reductase (PAO1 Δ*rmd* [OSA^+^ {OSA positive} CPA^−^ {CPA negative}]), (ii) epimerase (PAO1 Δ*wbpM* [OSA^−^ CPA^+^]), (iii) OSA polymerase (PAO1 Δ*wzy* [OSA^−^ CPA^+^]), (iv) common initiating glycosyltransferase (PAO1 Δ*wbpL* [OSA^−^ CPA^−^]), and (v) O-antigen ligase (PAO1 Δ*waaL* [OSA and CPA were still made but not attached to LPS in the periplasm]). In addition, we made mutants in the long (PAO1 Δ*wzz1* [OSA^+^ CPA^+^]) and very long (PAO1 Δ*wzz2* [OSA^+^ CPA^+^]) OSA chain length regulators (40, 41) (Fig. S3). We then determined the aggregate assembly type of the O-antigen mutants in SCFM2. We found that *ssg*, *wbpL*, and *wbpM* mutants with no OSA formed clumped aggregates, but the lack of CPA alone (PAO1 Δ*rmd*) did not change the aggregate assembly type (Fig. 2A and B).

10.1128/mBio.00860-21.3 FIG S3 The loss of *ssg* function changes the LPS OSA profile in P. aeruginosa. (A) Evolved isolates of PAO1 with a 1-bp deletion in *ssg* had the same OSA profile as that of PAO1 Δ*ssg*, and complementation of *ssg* in *trans* restored the OSA pattern. (B) The loss of *ssg* resulted in the loss of OSA and a change in the capped core pattern. Download FIG S3, TIF file, 0.6 MB. Copyright © 2021 Azimi et al. 2021 Azimi et al. https://creativecommons.org/licenses/by/4.0/ This content is distributed under the terms of the Creative Commons Attribution 4.0 International license.

**FIG 3:**
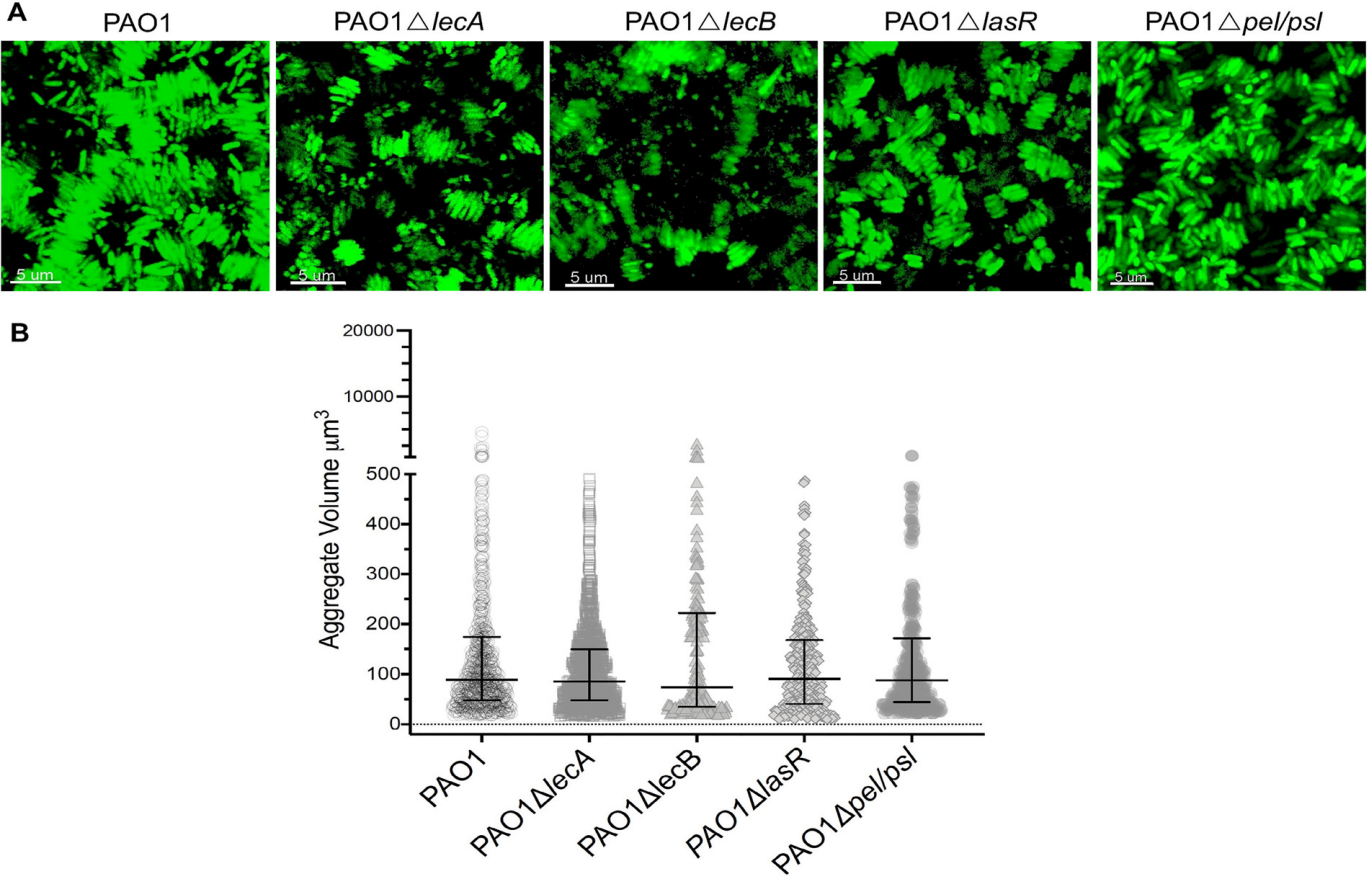
The aggregate assembly type is independent of exopolysaccharide production, lectins, and quorum sensing. (A) Loss of lectins (Δ*lecA* and Δ*lecB*), quorum sensing (Δ*lasR*), and exopolysaccharide components (Δ*pel* Δ*psl*) did not change the aggregate assembly type, and aggregates were assembled in stacked forms similar to those seen in PAO1. (B) Stacked aggregates formed by cells lacking lectins (Δ*lecA* and Δ*lecB*), quorum sensing (Δ*lasR*), and exopolysaccharide components (Δ*pel* Δ*psl*) were the same size as PAO1 aggregates (*P *= 0.1, *P* = 0.6, *P > *0.999, and *P > *0.999 by Kruskal-Wallis and Dunn’s multiple-comparison tests when aggregate volumes of Δ*lecA*, Δ*lecB*, Δ*lasR*, and Δ*pel* Δ*psl* cells were compared to those of PAO1; error bars are median aggregate volumes with interquartile ranges).

Biofilm formation by P. aeruginosa is regulated by several well-described mechanisms such as exopolysaccharide production, adhesins, and quorum sensing (QS) (6, 9, 42, 43). To determine whether any of these factors interfered with stacked aggregation in SCFM2, we examined the aggregate assembly of defined mutants in exopolysaccharide production (PAO1 Δ*pel* Δ*psl*) and lectins (PAO1 Δ*lecA* and PAO1 Δ*lecB*) and a mutant lacking a major QS regulator (PAO1 Δ*lasR*). Although the role of QS and biofilm formation remains controversial, we assessed this mutant because LasR regulates several pathways that could impact the aggregation type (7, 44). We found that all these mutants displayed stacked aggregate assemblies like PAO1 (Fig. 3). This indicated that changes in the aggregate assembly type were not due to alterations in common phenotypes associated with biofilm formation; only the loss of OSA was important.

### Loss of OSA leads to clumped aggregates, independent of polymer and cell density.

To determine differences in stacked and clumped aggregate assemblies in SCFM2, despite the presence of polymers, we monitored aggregate assemblies of PAO1 and PAO1 Δ*wbpL* over time. We found that an increase in the initial cell density resulted in the rapid formation of stacked aggregates, while there were no changes in clumped aggregate assembly (Movies S1 and S2).

10.1128/mBio.00860-21.8 MOVIE S1 Increased cell density reduced the time of stacked aggregation. To determine whether stacked aggregate assembly was dependent on cell density, we monitored the growth of PAO1 over time (initial density adjusted to an OD_600_ of 0.1 in 400 μl of SCFM2). Stacked aggregates were assembled after 180 min of growth. Download Movie S1, AVI file, 2.4 MB. Copyright © 2021 Azimi et al. 2021 Azimi et al. https://creativecommons.org/licenses/by/4.0/ This content is distributed under the terms of the Creative Commons Attribution 4.0 International license.

10.1128/mBio.00860-21.9 MOVIE S2 Increased cell density does not impact the timing of the formation of clumped aggregates. We monitored the growth of PAO1 Δ*wbpL* (initial density adjusted to an OD_600_ of 0.1 in 400 μl of SCFM2). There was no change in aggregate assembly over time. Download Movie S2, AVI file, 3.3 MB. Copyright © 2021 Azimi et al. 2021 Azimi et al. https://creativecommons.org/licenses/by/4.0/ This content is distributed under the terms of the Creative Commons Attribution 4.0 International license.

**FIG 4:**
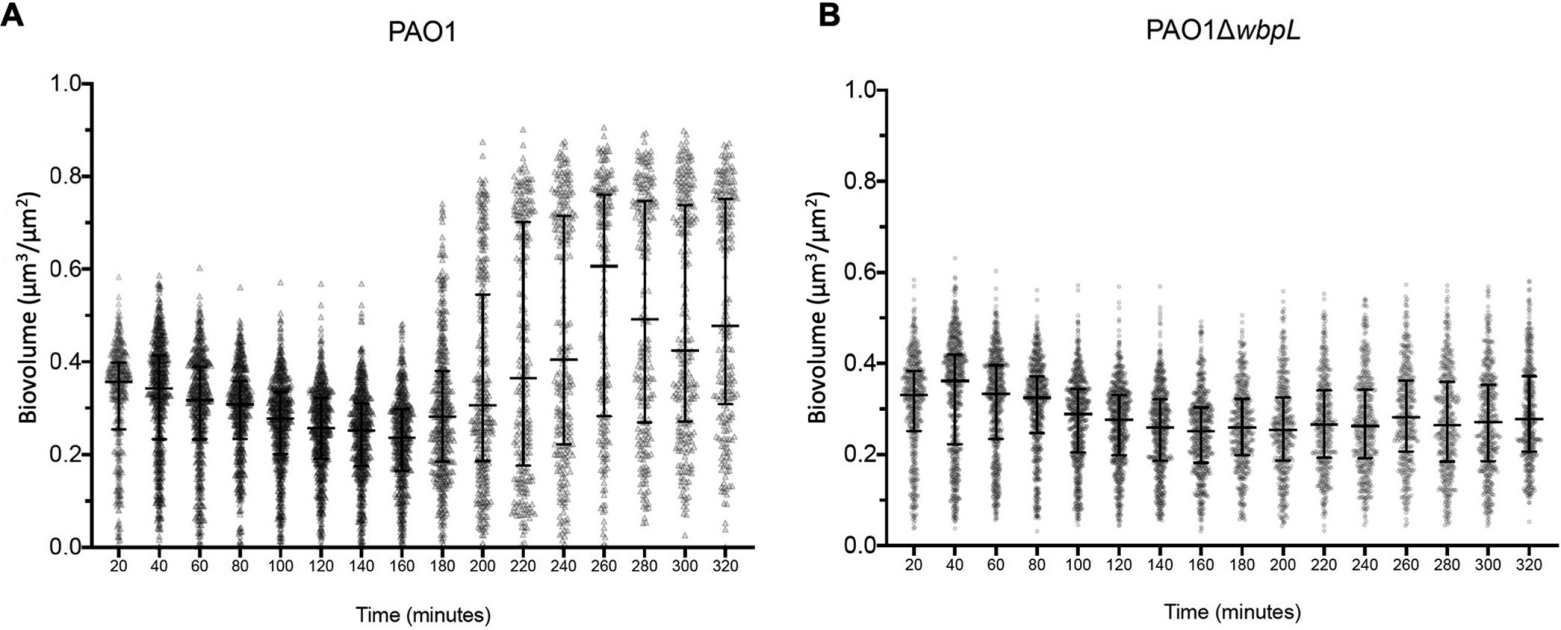
Clumped aggregate assembly is not dependent on cell density. (A) The aggregate biovolume of PAO1 significantly increased after 180 min of growth (median biovolume of 0.34 to 0.75 over time). (B) In PAO1 Δ*wbpL* (lacking OSA), the biovolume remained the same over time (median biovolume of 0.33 to 0.27 over time).

When we monitored the aggregate formation of PAO1 and PAO1 Δ*wbpL* over 6 h, we found that there was a significant change in the PAO1 aggregate biovolume after 180 min of cell growth in SCFM2 and when the stacks were assembled (Fig. 4A), whereas regardless of cell density, the biovolume of PAO1 Δ*wbpL* aggregates remained constant over time (Fig. 4B). We also found that reducing the concentration of both polymers in SCFM2 led to the dissolution of stacked aggregates, as expected, while it did not affect the formation of PAO1 Δ*wbpL* clumped aggregates (Fig. S4). These findings suggest that the loss of OSA prevents entropically derived stacked aggregate assembly, and the associated mechanism is independent of the polymer concentration and/or cell density. This is a well-studied manifestation of the aggregation of hydrophobic particles in colloidal environments (45, 46).

10.1128/mBio.00860-21.4 FIG S4 Stacked aggregate assembly is dependent on the concentrations of both eDNA and mucin. (A) Stacked aggregate formation was disrupted by diluting polymers in SCFM2. (B) Clumped aggregate assembly was independent of the polymer concentration in the environment. Download FIG S4, TIF file, 1.8 MB. Copyright © 2021 Azimi et al. 2021 Azimi et al. https://creativecommons.org/licenses/by/4.0/ This content is distributed under the terms of the Creative Commons Attribution 4.0 International license.

### Aggregate assembly of P. aeruginosa is not serotype specific but dependent on cell surface relative hydrophobicity.

**FIG 5:**
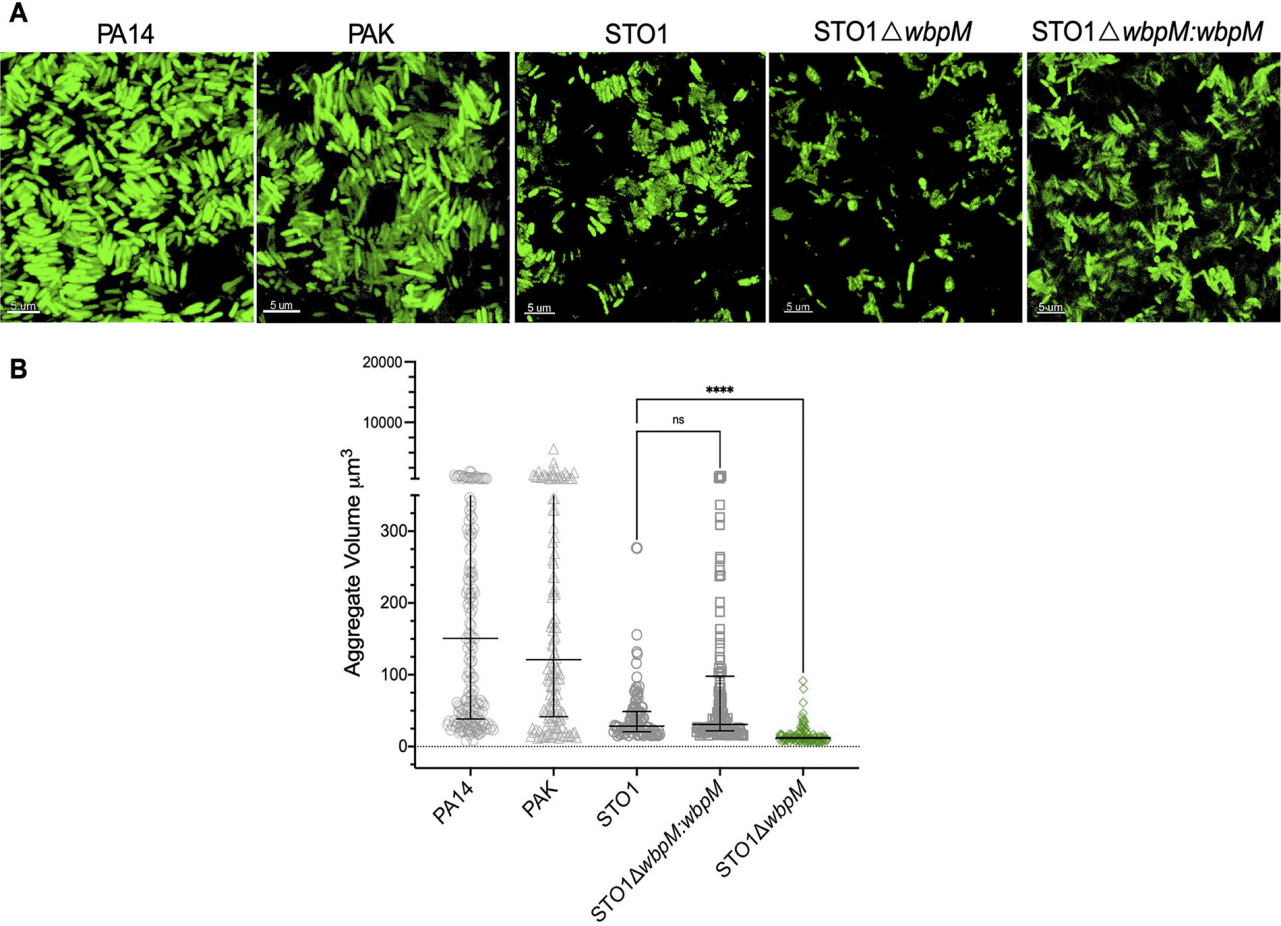
Clumped aggregate assembly of P. aeruginosa is not serotype specific. (A) P. aeruginosa PA14, PAK, and STO1 formed stacked aggregates in SCFM2, and OSA^−^ mutant STO1 (STO1 Δ*wbpM*) led to a clumped assembly of aggregates. (B) The loss of OSA altered aggregate assembly from stacked to clumped in STO1 and significantly decreased the aggregate volume (*P* = 0.0048 by Kruskal-Wallis and Dunn’s multiple-comparison tests; error bars are median aggregate volumes with interquartile ranges, and each data point is representative of an aggregate).

There are 20 serotypes of P. aeruginosa based on the glycosyl groups of OSA (38). As our findings were limited to PAO1 (serotype O5), we examined the aggregate formation of PA14 (serotype O10), PAK (serotype O6), and STO1 (serotype O1), which all differ in the oligosaccharide units of OSA (38). In all serotypes, we observed a stacked assembly similar to that of PAO1, but in an STO1 strain lacking OSA (Δ*wbpM*), we identified small clumped aggregates, with the restoration of stacks when *wbpM* was complemented in *trans* (Fig. 5A and B). These data confirm that the aggregate assembly type is not serotype specific.

**FIG 6:**
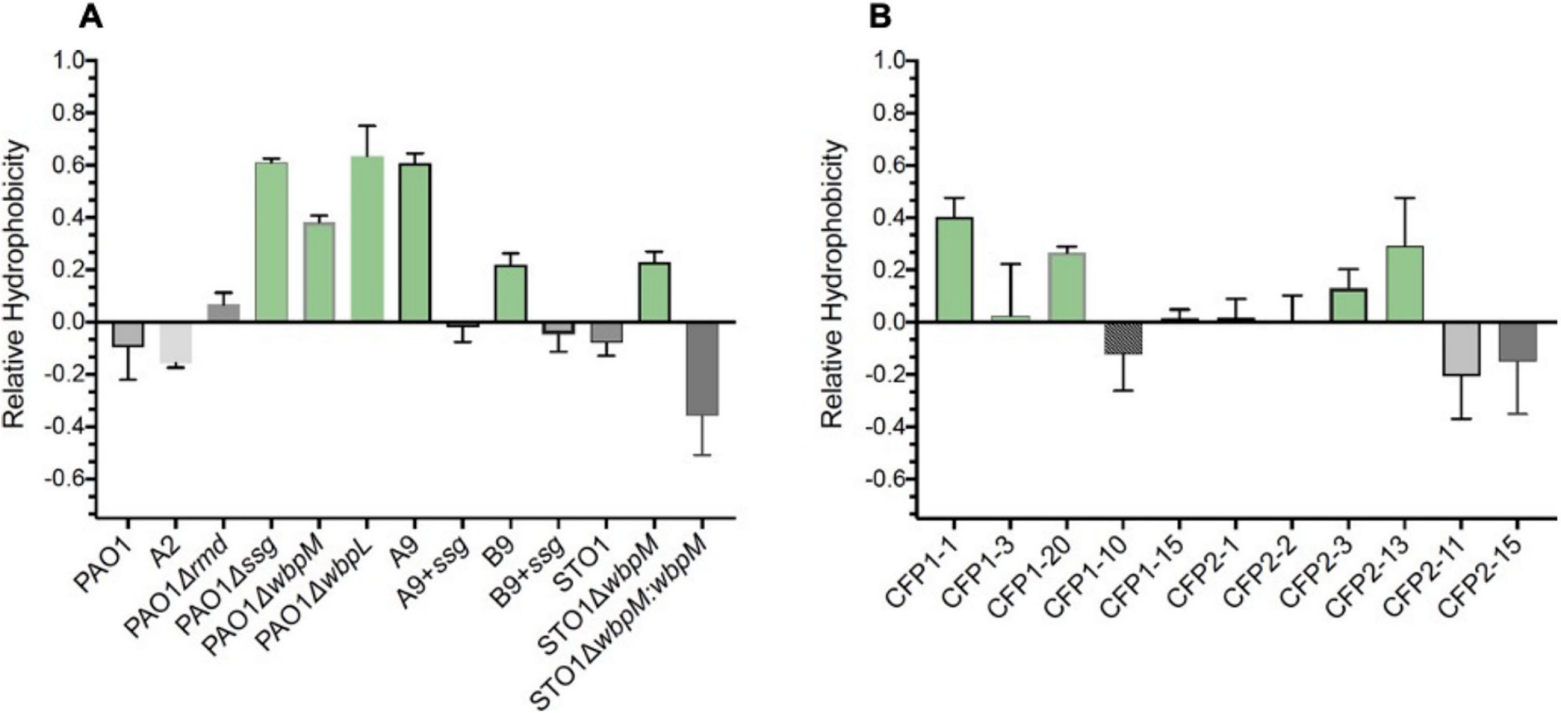
Cell surface hydrophobicity determines the aggregate assembly type. (A) The relative cell surface hydrophobicity was dependent on OSA, and mutations in *ssg*, *wbpL*, and *wbpM* led to an increase in the relative hydrophobicity of PAO1 and STO1 (green bars). (B) There was heterogeneity in the relative hydrophobicity of cell surfaces of P. aeruginosa isolates collected from two CF expectorated sputum samples (CFP1 and CFP2).

Previously, it has been shown that the lack of OSA increases the hydrophobicity of the P. aeruginosa cell surface (30). To determine whether hydrophobicity correlated with aggregate type, we assessed the relative surface hydrophobicity of OSA^+^ and OSA^−^ strains. We found a significant increase in surface hydrophobicity in OSA mutants (Fig. 6A), which corresponded with a clumping aggregate phenotype (Fig. 2). The loss of OSA is a common adaptive trait of P. aeruginosa in CF airways, possibly leading to an increase in cell surface hydrophobicity that could alter the spatial organization of P. aeruginosa cells in CF airways. We therefore evaluated the relative hydrophobicity of 11 P. aeruginosa isolates collected from the expectorated sputa of 2 individuals with CF. We observed heterogeneity in the cell surface relative hydrophobicity of the CF isolates across the two patients (Fig. 6B).

## DISCUSSION

Despite a large body of work showing how P. aeruginosa adapts to the CF lung environment (19–21, 33, 47, 48) and population heterogeneity during chronic infection (20, 21, 23, 49, 50), little is known about the impact of this intraspecific heterogeneity on the formation of P. aeruginosa aggregates. To test whether genetic heterogeneity impacts aggregation, we investigated the aggregate formation of selected evolved isolates from a previous 50-day biofilm evolution experiment of PAO1 (23) in SCFM2 (24). We found that (i) there are two distinct types of aggregate assemblies formed by P. aeruginosa in SCFM2, (ii) the OSA impacts the aggregate assembly type, and (iii) the loss of OSA and LPS core +1 increases the hydrophobicity of the bacterial surface, which prevents depletion aggregation.

Previously, it was shown that the aggregation of P. aeruginosa cells in a polymer-rich environment can be due to depletion forces, where the reduction of the free energy by increased entropy of the whole system leads to stacked aggregation of bacterial cells (27). The change of the polymer electrostatic properties in the same study altered the aggregate assembly to bridging assembly, suggesting that the aggregate assembly type is driven by physical properties of the environment and that the biological properties of the cells assume little or no role in the aggregate assembly type (27). However, we observed two distinct types of aggregate assemblies by genetically diverse PAO1 isolates in SCFM2. Stacked aggregation is dependent on the cell density and polymer concentration, and the increase in entropy is the driving force behind these assemblies. In contrast, clumping assembly is driven by changes in the surface properties of P. aeruginosa cells. This indicates that although aggregate formation is influenced by physical forces, OSA strongly impacts the spatial organization enforced by the physical properties of the environment.

Other studies have suggested that during CF infection, polymers like mucin can disperse cells in established biofilms (51), although our work suggests that polymers are more likely to influence the spatial arrangement of the cells. The clumping aggregate assembly was not influenced by factors that have previously been shown to be involved in biofilm and aggregate formation, including lectins (42, 52), QS (6, 9, 53), and EPS (54–57). While we found that stacked aggregates had a larger biovolume than clumped aggregates and that this can be explained by depletion aggregation, other explanations also exist. First, stacked aggregates might be more likely to support growth within the aggregate, leading to larger aggregates. Second, as clumped aggregates become larger, they may be more prone to disaggregation, leading to them never achieving a larger size even if there was cellular division occurring in the aggregate.

It remains to be determined whether stacked aggregates are found during human CF infection, although a recent study demonstrated that in CF airways, eDNA surrounds aggregates, suggesting that in CF lungs, eDNA could increase entropic force and form aggregates via depletion aggregation (58). It is noteworthy that stacked aggregates form in other species of bacteria such as Rhizobium leguminosarum, which was dependent on LPS core and O-antigen (59). Furthermore, honeycomb structures of P. aeruginosa (multilayers of stacks) were shown to form in the crops of Drosophila melanogaster, highlighting that stacked aggregates are found in some types of infection (60).

By assessing the relative hydrophobicity of cells forming different aggregate types, we found that P. aeruginosa cells that form clumped aggregates have surfaces with higher relative hydrophobicity. In agreement with previous studies (29, 61–63), we showed that this was due to a loss of OSA and the exposure of uncapped LPS core. Regulating the aggregate assembly type by directly altering OSA expression levels in environments containing differential levels of cells and polymers (such as CF sputum) may allow cells to better resist environmental stressors such as the host immune response, antibiotics, or phage. The loss of OSA has been reported in P. aeruginosa strains isolated from CF sputum, suggesting that finding small, clumped aggregates in CF sputum and CF airways (64) might be directly due to the loss of OSA. As our work was performed *in vitro*, further studies are required to determine how OSA and surface hydrophobicity impact aggregate formation in P. aeruginosa isolates growing in CF lungs.

Overall, our findings highlight that the surface properties of P. aeruginosa cells determine how they form aggregates in environments with different physicochemical properties, providing potential benefits from social interactions with highly related cells. Our findings also highlight that changes in the cell surface properties may influence how aggregates form in other species of bacteria and provide explanations as to how different P. aeruginosa strains and species can stably coexist in microbiomes.

## MATERIALS AND METHODS

### Bacterial strains and culture conditions.

We selected 7 evolved isolates of PAO1 from 50-day-evolved populations in SCFM (23). We transformed all P. aeruginosa strains used in this study with the pME6032:*gfp* plasmid (65) using electroporation (66). Briefly, to prepare electrocompetent P. aeruginosa cells, we grew the bacterial cells in LB broth overnight, washed the cultures grown overnight with a 300 mM sucrose solution at room temperature, and then resuspended the bacterial pellets in 1 ml of 300 mM sucrose. We then electroporated 50 μl of electrocompetent cells with 2 μl of purified plasmid and recovered the cells by the addition of 950 μl of LB broth and incubation at 37°C at 200 rpm for 30 min. We selected the transformed cells by plating out the electroporated bacteria onto LB agar plates supplemented with 200 μg/ml of tetracycline. We obtained the clinical isolates from the Emory CF@LANTA Research Center. Patients in this study were aged between 21 and 29 years at the time of collection of the sputum samples. This study was approved by the Institutional Review Boards (IRBs) at the Georgia Institute of Technology and Emory University Hospital. A list of all bacterial strains used in this study is available in Table S2 in the supplemental material.

10.1128/mBio.00860-21.6 TABLE S2 List of the strains used in this study. Download Table S2, DOCX file, 0.01 MB. Copyright © 2021 Azimi et al. 2021 Azimi et al. https://creativecommons.org/licenses/by/4.0/ This content is distributed under the terms of the Creative Commons Attribution 4.0 International license.

### Determining diversity in colony morphologies.

To determine diversity in colony morphology in evolved populations (23), we used a Congo red-based agar medium (1% agar, 1× M63 salts [3 g monobasic KHPO_4_, 7 g K_2_PO_4_, and 2 g NH_4_·2SO_4_, with the pH adjusted to 7.4], 2.5 mM magnesium chloride, 0.4 mM calcium chloride, 0.1% Casamino Acids, 0.1% yeast extract, 40 mg/liter Congo red solution, 100 μM ferrous ammonium sulfate, and 0.4% glycerol) (67). We inoculated each evolved isolate in LB broth, incubated the colony for 6 h at 37°C at 200 rpm, and then spotted 10 μl of the culture onto Congo red agar plates. We incubated the plates at 37°C for 24 h and a further 4 days at room temperature.

### Genomic DNA extraction and whole-genome sequencing.

We plated each of the selected evolved isolates on LB agar plates, picked single colonies of each isolate, inoculated the colonies in 5 ml of SCFM (35), and incubated the isolates overnight at 37°C at 200 rpm. We extracted the genomic DNA using the Qiagen DNeasy blood and tissue kit. We prepared sequencing libraries using the Nextera XT protocol (Illumina) and sequenced the libraries in a 24-plex format on the Illumina MiSeq platform to obtain an approximate calculated level of coverage of 50× for each evolved isolate. For single nucleotide polymorphism (SNP) calling, we used breseq analysis (consensus mode) (37, 68, 69) and compared the genetic variation in each evolved isolate to the PAO1 ancestral strain.

### Image acquisition and analysis.

For imaging aggregates in SCFM2 (24), we inoculated each bacterial isolate into tryptic soy broth (TSB) supplemented with 200 μg/ml of tetracycline and incubated the mixture at 37°C at 200 rpm overnight. We inoculated 50 μl of the culture grown overnight into 5 ml of SCFM and incubated the culture at 37°C at 200 rpm for 5 to 6 h until the cultures reached mid-log phase (optical density at 600 nm [OD_600_] = 0.5). We then adjusted the OD_600_ to 0.05 in 400 μl of freshly made SCFM2 containing 0.6 mg/ml of DNA and 5 mg/ml of mucin (8, 24). We incubated the cultures at 37°C for 16 h in chamber slides (Lab-Tek) before image acquisition. We used an LSM880 confocal microscope equipped with a 63× oil immersion lens for image acquisition, scanned the aggregates using a diode laser at 488 nm, and collected the fluorescence emission between 480 and 530 nm for image acquisition. For imaging the cells grown in SCFM, we adjusted the OD_600_ of cells from mid-log-phase growth to 0.05 in 400 μl of freshly made SCFM. We incubated the cultures at 37°C for 16 h in chamber slides before image acquisition. For image analysis, we used Imaris 9.0.1 image analysis software to analyze the morphological properties of the aggregates and measured the surface area and volume of each aggregate using a surface model algorithm. We used the same parameters for particle and voxel sizes. We measured the aggregate volume and surface area in 10 images acquired for each strain in three independent experiments (over 1,000 aggregates were measured in total under each condition). For time course experiments, we used the same image acquisition parameters, using the time series option and imaging as Z stacks every 20 min for up to 10 h. To assess the role of bacterial cell density in aggregation, we adjusted the OD_600_ to 0.1 in 400 μl of SCFM2 and imaged the cells every 20 min for 6 h. We prepared time series videos using the three-dimensional (3D) plug-in in Fiji (70) and Adobe Lightroom.

### Gene deletion and complementation.

We used standard genetic techniques for the construction of P. aeruginosa mutants. To delete *ssg*, *rmd*, *wbpL*, *wbpM*, *waaL*, *wzy*, *wzz1*, and *wzz2*, we PCR amplified 600-bp DNA sequences flanking the open reading frame of each gene using Q5 DNA polymerase (New England BioLabs). We then cloned these sequences into EcoRI-XbaI-digested pEXG2 by Gibson assembly using NEBuilder HiFi assembly master mix (New England BioLabs) and transformed them into Escherichia coli S17 λ*pir*. We verified cloned inserts by colony PCR and Sanger sequencing (Eurofins Genomics). We introduced the deletion constructs into PAO1 by electroporation and selected strains carrying single-crossover insertions of the deletion constructs on LB agar plates supplemented with 100 μg/ml gentamicin. We cultured gentamicin-resistant colonies in LB without antibiotic and plated the colonies on LB agar plates with 0.25% NaCl and 5% sucrose. We then selected sucrose-resistant colonies, screened them for gentamicin sensitivity to ensure the loss of the pEXG2 construct, and assessed them for the desired gene deletion by colony PCR and Sanger sequencing of the PCR product. For *ssg* complementation, we PCR amplified the *ssg* coding sequence and 100 bp of upstream sequence (including the *ssg* native promoter) using Q5 DNA polymerase (New England BioLabs). We cloned this 1,057-bp product into KpnI-BamHI-digested pUC18T-miniTn7-Gent by Gibson assembly using NEBuilder HiFi assembly master mix (New England BioLabs) and transformed it into E. coli S17 λ*pir*. We verified the cloned insert by colony PCR and Sanger sequencing (Eurofins Genomics). We cotransformed the complementation construct with the Tn*7* helper plasmid pTNS3 into PAO1 Δ*ssg*, evolved isolates by electroporation, and selected isolates on LB agar plates supplemented with 100 μg/ml gentamicin. We verified the strains for *ssg*^+^ complementation by colony PCR and for the loss of the pUC18-miniTn7-Gent vector and pTNS3 by screening for carbenicillin sensitivity.

### LPS extraction.

We isolated bacterial lipopolysaccharide by the hot phenol extraction method (71). Briefly, we pelleted 5 ml of cultures of PAO1 and PAO1-derived strains grown overnight in LB broth by centrifugation for 10 min at 4,200 × *g*. We resuspended the pellets in 200 μl 1× SDS buffer (2% β-mercaptoethanol [BME], 2% SDS, 10% glycerol, 50 mM Tris-HCl [pH 6.8]) and incubated them at 99°C for 15 min. Next, we added 5 μl of 20 mg/ml proteinase K (Sigma) to each tube and incubated the cell lysates at 59°C for 3 h. Next, we added 200 μl of ice-cold Tris-saturated phenol to each sample, vortexed the mixture for 10 min, added 1 ml diethyl-ether, and vortexed the mixture for a further 10 s. We centrifuged the samples for 10 min at 16,000 × *g* and extracted the bottom layer. We performed a second extraction with phenol and diethyl-ether as described above. We mixed an equal volume of the extracted LPS samples with an equal volume of 2× SDS buffer and electrophoresed 10 μl of each sample on Novex 4 to 20% polyacrylamide gradient gels (Thermo Fisher) in Tris-glycine-SDS buffer. Following electrophoresis, we visualized LPS with a ProQ Emerald lipopolysaccharide staining kit (Thermo Fisher).

### Assessing cell surface hydrophobicity.

To assess the levels of cell surface hydrophobicity, we used hydrophobic interaction chromatography (29). Briefly, we grew bacterial cells for 6 to 8 h at 37°C at 200 rpm to reach mid-log phase. We harvested the cells, washed the cells three times with ice-cold 3 M NaCl (pH 7), and resuspended the cells in 3 M NaCl. We used octyl-Sepharose CL-4C beads (Sigma) to assess the interaction of hydrophobic cells with these beads compared to control Sepharose CL-4C beads (Sigma). We prepared bead columns by three washes of the beads with Milli-Q water and then three washes with 3 M NaCl (pH 7) (at 4°C). We then prepared 1-ml columns of both beads by using 3-mm-diameter filter paper. We added 100 μl of the bacterial suspension and incubated the mixture at room temperature for 15 min. We measured the OD_450_ of the flowthrough from each column. We calculated the relative hydrophobicity based on the ratio of the OD_450_ octyl-Sepharose CL-C4 column flowthrough to that of the control column.

### Statistical analysis.

For statistical analysis of the aggregate volume and biovolume distribution, we used GraphPad Prism 8.0.

### Data availability.

The sequences in this study are available at the NCBI SRA database (accession number PRJNA702741).
