# Supplementary figures and images for: O-Specific Antigen-Dependent Surface Hydrophobicity Mediates Aggregate Assembly Type in Pseudomonas aeruginosa
Source: mBio. 2021 Aug 10;12(4):e00860-21. doi: 10.1128/mBio.00860-21 (PMC8406328; doi:10.1128/mBio.00860-21)

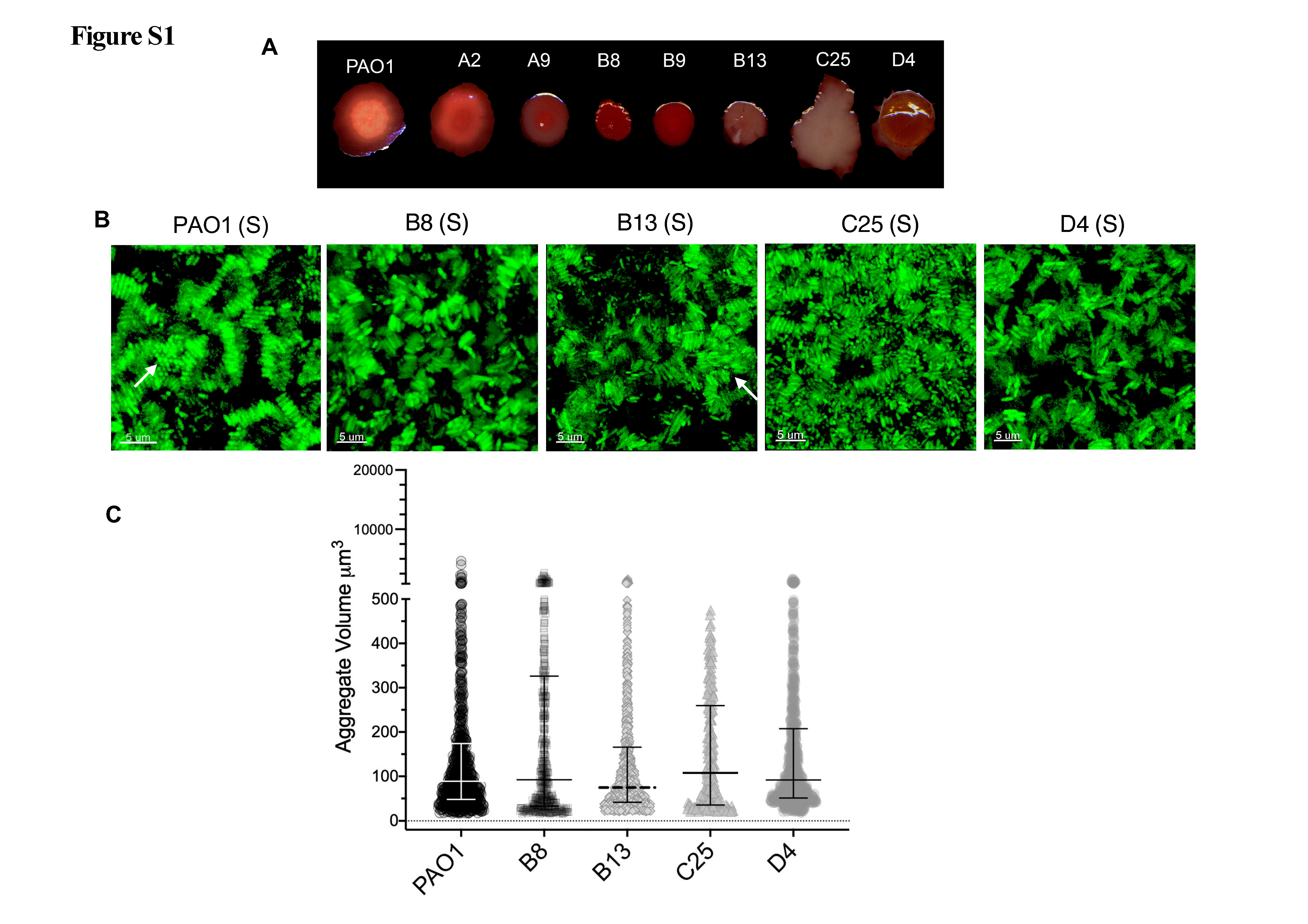

Supplement: FIG S1 [file mbio.00860-21-sf001.tif]

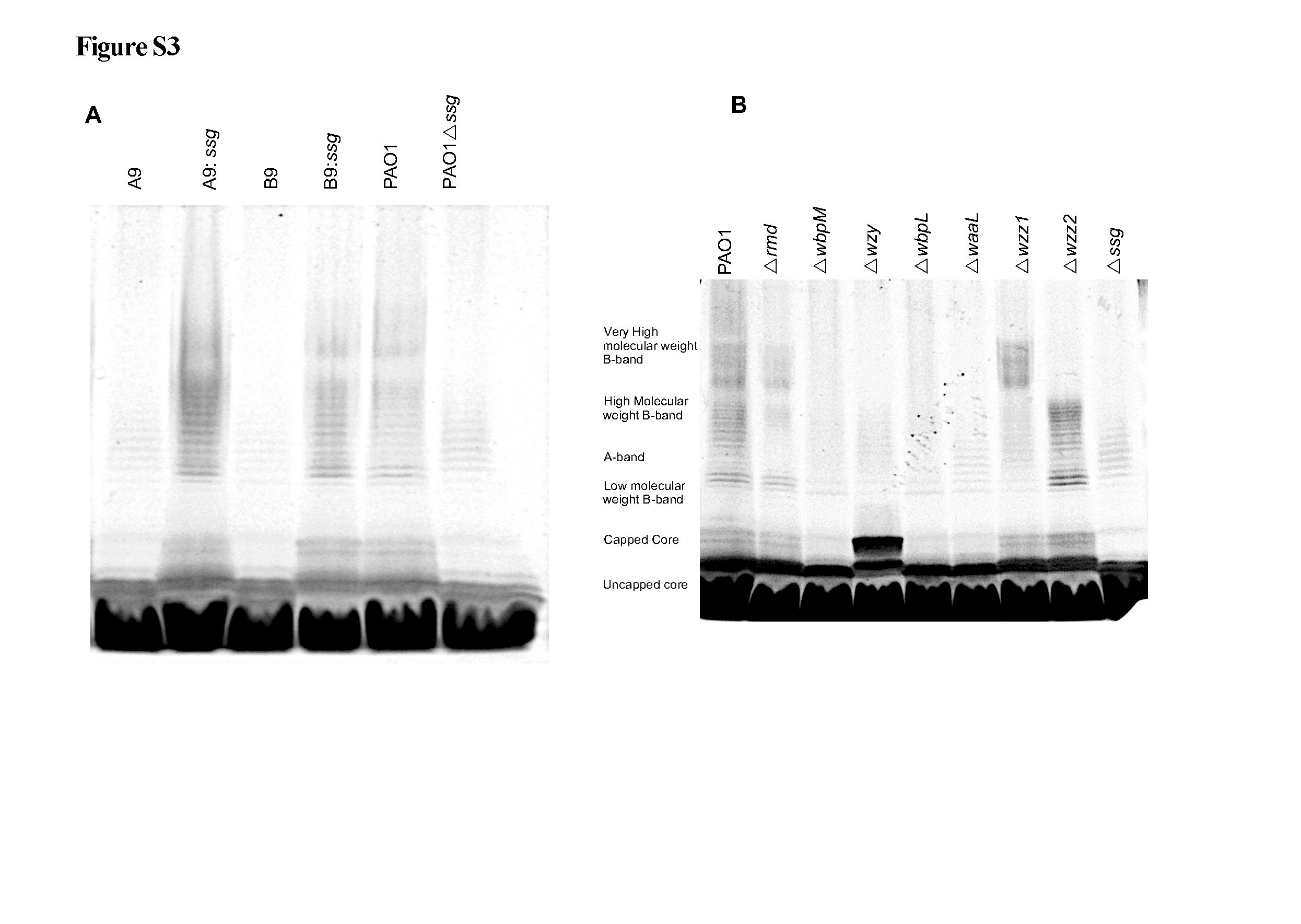

Supplement: FIG S3 [file mbio.00860-21-sf003.tif]

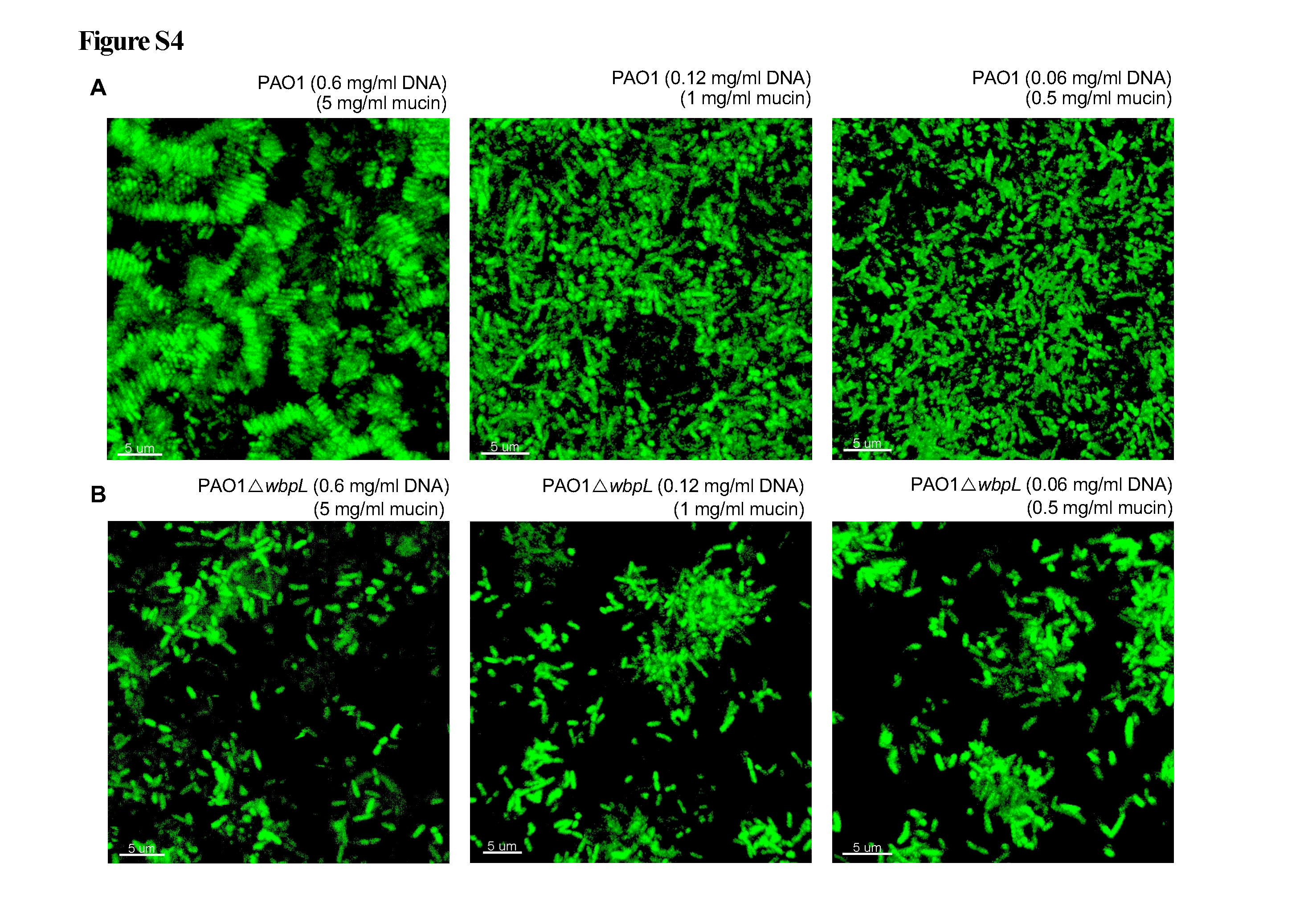

Supplement: FIG S4 [file mbio.00860-21-sf004.tif]
