# Supplementary material for: O-Specific Antigen-Dependent Surface Hydrophobicity Mediates Aggregate Assembly Type in Pseudomonas aeruginosa
Source: mBio. 2021 Aug 10;12(4):e00860-21. doi: 10.1128/mBio.00860-21 (PMC8406328; doi:10.1128/mBio.00860-21)
Supplement: TABLE S1 [file mbio.00860-21-st001.docx]

|  | **PAO1** | **A2** | **A9** | **B9** |
| --- | --- | --- | --- | --- |
| Minimum | 0.110031 | 0.12282713 | 0.100064 | 0.10049691 |
| 25% Percentile | 0.248399 | 0.267627 | 0.222612 | 0.15592075 |
| Median | 0.35255 | 0.312206 | 0.277916 | 0.2315546 |
| 75% Percentile | 0.41007625 | 0.393158 | 0.33127 | 0.33155867 |
| Maximum | 0.559253 | 0.600513 | 0.371328 | 0.469442558 |
