# Supplementary material for: O-Specific Antigen-Dependent Surface Hydrophobicity Mediates Aggregate Assembly Type in Pseudomonas aeruginosa
Source: mBio. 2021 Aug 10;12(4):e00860-21. doi: 10.1128/mBio.00860-21 (PMC8406328; doi:10.1128/mBio.00860-21)
Supplement: TABLE S2 [file mbio.00860-21-st002.docx]

| ***P. aeruginosa* strain** | **Description** |  | **Source** |
| --- | --- | --- | --- |
| **PAO1** | Wild type PAO1 (Nottingham strain) |  | Holloway collection |
| **A2** | Evolved PAO1 isolate | Evolved isolate | This study |
| **A9** | Evolved PAO1 isolate | Evolved isolate | This study |
| **A9:*ssg*** | Evolved PAO1 isolate complemented with *ssg* gene | Evolved isolate | This study |
| **B8** | Evolved PAO1 isolate | Evolved isolate | (1) |
| **B9** | Evolved PAO1 isolate | Evolved isolate | This study |
| **B9:*ssg*** | Evolved PAO1 isolate complemented with *ssg* gene | Evolved isolate | This study |
| **B13** | Evolved PAO1 isolate | Evolved isolate | This study |
| **C25** | Evolved PAO1 isolate | Evolved isolate | This study |
| **D4** | Evolved PAO1 isolate | Evolved isolate | This study |
| **PAO1Δ*ssg*** | PAO1 with *ssg* deletion | Isogenic mutant | This study |
| **PAO1Δ*rmd*** | PAO1 with *rmd* deletion/CPA^−^ |  | This study |
| **PAO1Δ*wbpM*** | PAO1 with *wbpM* deletion/OSA^−^ |  | This study |
| **PAO1Δ*wbpL*** | PAO1 with *wbpL* deletion/ CPA^−^ OSA^−^ |  | This study |
| **PAO1Δ*waal*** | PAO1 with *waal* deletion/ OSA^−^ |  | This study |
| **PAO1Δ*wzy*** | PAO1 with *wzy* deletion/ OSA^−^ |  | This study |
| **PAO1Δ*wzz1*** | PAO1 with *wzz1* deletion/No high molecular weight B-band |  | This study |
| **PAO1Δ*wzz2*** | PAO1 with *wzz2* deletion/No very high molecular weight B-band |  | This study |
| **STO1** | Clinical isolate of *P. aeruginosa*serotype O-1 |  | [2] |
| **PA14** | PA14 wildtype |  |  |
| **PAK** | PAK wildtype |  |  |
| **STO1Δ*wbpM*** | Clinical isolate of *P. aeruginosa* serotype O-1/ OSA^−^ |  | [2] |
| **STO1Δ*wbpM:wbpM*** | Clinical isolate of *P. aeruginosa* serotype O-1; OSA^−^ / complemented with serotype O-1 *wbpM* |  | [2] |
| **PAO1Δ*lecA*** | PAO1 with *lecA* deletion |  | [3] |
| **PAO1Δ*lecB*** | PAO1 with *lecB* deletion |  | This study |
| **PAO1Δ*pel/psl*** | PAO1 with *pel/psl* deletions |  | [4] |
| **PAO1Δ*lasR*** | PAO1 with *lasR* deletion |  | This study |
| **CFP1-1** | CF isolate of *P. aeruginosa, isolated from* CFP1 sputum sample | Clinical isolate | This study |
| **CFP1-3** | CF isolate of *P. aeruginosa, isolated from* CFP1 sputum sample | Clinical isolate | This study |
| **CFP1-10** | CF isolate of *P. aeruginosa, isolated from* CFP1 sputum sample | Clinical isolate | This study |
| **CFP1-15** | CF isolate of *P. aeruginosa, isolated from* CFP1 sputum sample | Clinical isolate | This study |
| **CFP1-20** | CF isolate of *P. aeruginosa, isolated from* CFP1 sputum sample | Clinical isolate | This study |
| **CFP2-1** | CF isolate of *P. aeruginosa, isolated from* CFP2 sputum sample | Clinical isolate | This study |
| **CFP2-2** | CF isolate of *P. aeruginosa, isolated from* CFP2 sputum sample | Clinical isolate | This study |
| **CFP2-3** | CF isolate of *P. aeruginosa, isolated from* CFP2 sputum sample | Clinical isolate | This study |
| **CFP2-11** | CF isolate of *P. aeruginosa, isolated from* CFP2 sputum sample | Clinical isolate | This study |
| **CFP2-13** | CF isolate of *P. aeruginosa, isolated from* CFP2 sputum sample | Clinical isolate | This study |
| **CFP2-15** | CF isolate of *P. aeruginosa, isolated from* CFP2 sputum sample | Clinical isolate | This study |

1. Azimi, S., et al., *Allelic polymorphism shapes community function in evolving Pseudomonas aeruginosa populations.* ISME J, 2020.

2. Davis, M.R., Jr., et al., *Identification of the mutation responsible for the temperature-sensitive lipopolysaccharide O-antigen defect in the Pseudomonas aeruginosa cystic fibrosis isolate 2192.* J Bacteriol, 2013. **195**(7): p. 1504-14.

3. Diggle, S. P., et al., *The galactophilic lectin, LecA, contributes to biofilm development in Pseudomonas aeruginosa*. Environ Microbiol, 2006. **8**(6): p. 1095-1104.

4. Irie, Y., et al., *The Pseudomonas aeruginosa PSL Polysaccharide Is a Social but Noncheatable Trait in Biofilms.* mBio, 2017. **8**(3).
